# PROMISE—impact of maternal and neonatal risk factors on the respiratory outcome of extremely preterm infants following PPROM in the second trimester of pregnancy

**DOI:** 10.3389/fped.2026.1776970

**Published:** 2026-04-08

**Authors:** C. Brickmann, N. Gauger, J. Hauer, M. Kunze, O. Neumann, C. Scholz, S. Loth, M. Krüger

**Affiliations:** 1 Technical University of Munich, TUM School of Medicine and Health, Department of Pediatrics, Kinderklinik Muenchen Schwabing, TUM University Hospital, Munich, Germany; 2 Clinic for Neonatology, Muenchen Klinik, Munich, Germany; 3 University of Freiburg, Department of Gynecology and Obstetrics, University Hospital Freiburg, Freiburg, Germany; 4 Clinic for Gynaecology and Obstetrics, München Klinik Schwabing, Munich, Germany; 5 Clinic for Gynaecology and Obstetrics, München Klinik Harlaching, Munich, Germany

**Keywords:** dry lung, PPROM (preterm premature rupture of membranes), prenatal counselling, pulmonary hypoplasia, respiratory outcome, VLBW (very low birth weight)

## Abstract

**Background:**

Prolonged preterm premature rupture of membranes (PPROM) in the second trimester is associated with heterogeneous neonatal respiratory outcomes, ranging from mild transitional respiratory impairment to dry lung syndrome (DL), pulmonary hypoplasia (PH), bronchopulmonary dysplasia (BPD) and death. Risk assessment is challenging because outcomes are influenced by both gestational maturity and PPROM-specific exposures. This study aimed to evaluate respiratory outcomes after prolonged PPROM using a multifactorial and longitudinal approach that integrates gestational age, amniotic fluid status, inflammatory signs, and maternal characteristics.

**Methods:**

We retrospectively analyzed 66 pregnancies with second-trimester PPROM and prolonged latency. Short-term respiratory outcomes were classified as no respiratory disease, dry lung (DL), or pulmonary hypoplasia (PH); neonatal death and BPD were also assessed. Gestational age (GA) at PPROM and at birth were analyzed using ordered groupings with trend testing. Multivariable logistic regression was used to identify independent risk factors. Serial weekly measurements of the single deepest vertical pocket (SDP) were evaluated longitudinally using repeated-measures analysis to compare amniotic fluid trajectories between outcome groups.

**Results:**

Respiratory morbidity and death decreased stepwise with increasing GA at PPROM and GA at birth. Lower SDP values were consistently associated with PH, BPD, and death, particularly when median SDP was <2 cm. Longitudinal analysis demonstrated persistently lower SDP values in pregnancies resulting in PH, without evidence of differing SDP trajectories over time between groups. Inflammatory signs were associated with BPD, while maternal age and GA at PPROM were associated with PH and death. DL showed a distinct pattern, with weaker associations to gestational age and amniotic fluid parameters compared with PH.

**Conclusion:**

Outcomes after prolonged second-trimester PPROM reflect a multifactorial risk profile in which gestational maturity, amniotic fluid impairment, inflammatory status, and maternal characteristics interact. Integrating longitudinal ultrasound data with gestational-age–based risk stratification can improve counseling and support individualized management strategies in PPROM pregnancies.

## Introduction

Preterm premature rupture of membranes (PPROM) presents not only a common cause of preterm birth but also an ongoing challenge in predicting neonatal outcome (1–3). PPROM, particularly in the mid-trimester before 32 weeks of gestation, remains a critical issue in perinatal medicine, contributing significantly to neonatal morbidity and mortality (4, 5). Despite advances in prenatal and neonatal care, predicting neonatal outcomes in these cases remains a major challenge (6). PPROM may lead to extreme prematurity, the development of dry lung syndrome (DL), severe pulmonary hypoplasia (PH), or, in some cases, surprisingly favorable outcomes with minimal complications. At the severe end of prolonged oligo-/anhydramnios, complications may include Potter sequence (facial and limb deformities) and skeletal abnormalities due to fetal constraint. The variability makes counseling and management complex for clinicians and families alike (7). Our analysis focuses on neonatal respiratory outcomes and survival in pregnancies with second-trimester PPROM and prolonged latency managed in tertiary care.

PPROM affects approximately 3% of pregnancies and precedes up to 35% of preterm births (4). Earlier gestational age (GA) at PPROM is generally associated with longer latency intervals before delivery and critically influences outcomes, with over 50% of cases delivering within seven days. Yet, even with known risk factors such as GA at rupture, latency duration, and degree of oligohydramnios, accurately forecasting individual neonatal outcomes—especially the development of PH or DL—remains elusive (6–11).

Pulmonary hypoplasia, one of the most feared complications, results from prolonged oligohydramnios during the canalicular phase of lung development (16–28 weeks). Its incidence varies widely (1%–48%), reflecting inconsistencies in diagnostic criteria and definitions (8). DL syndrome, a distinct entity, represents functional airway collapse that can mimic true hypoplasia but may show rapid postnatal improvement (12, 13). Distinguishing between these conditions antenatally remains a diagnostic challenge (14).

Management strategies for PPROM are heterogeneous and continue to evolve. While initial inpatient health monitoring and treatment of mother and fetus with i.e., corticosteroids and antibiotics is standard, outpatient management is considered for select stable patients (4, 15). The effectiveness and safety of outpatient care are debated, and its impact on neonatal outcomes, particularly the risk of PH or DL, is unclear. Interventions such as amnioinfusion aim to restore amniotic fluid volume and reduce pulmonary risks, but robust evidence from randomized trials is lacking (16–18). Overall, expectative management is considered as thought to be most effective (4, 19).

Infection and inflammation further complicate the picture, as both are associated with adverse neonatal outcomes, yet early detection remains problematic (7, 19–22). The role of the maternal vaginal microbiome and specific pathogens like Group B Streptococcus (GBS) is well established in perinatal infection, but their impact on predicting severe pulmonary outcomes is not yet fully understood (23, 24).

Population-based studies have highlighted that survival and intact neurodevelopment are possible, even in extreme prematurity following PPROM (19). However, predicting which neonate will experience severe complications like PH or DL remains difficult, underscoring the need for improved predictive tools and biomarkers (1, 8, 10, 21). Currently, there are no established guidelines to assist clinicians in weighing the risks of pregnancy prolongation vs. accepting preterm delivery.

The PROMISE (Preterm Rupture Outcomes: Maternal, Inflammatory, and Sonographic Evaluation) Study aims to provide further insights into maternal and neonatal risk factors that influence the wide range of outcomes seen after mid-trimester PPROM with prolonged latency. We hope to support evidence-based counseling and refine management strategies for these complex and high-risk pregnancies.

## Methods

### Study design and population

This study was conducted as a retrospective cohort analysis at two Level-III perinatology centers in Germany. The observation period spanned from January 1, 2018, to December 31, 2024. A total of 66 neonates with a history of PPROM during the second trimester were included in the analysis. The primary objective of the study was to evaluate the impact of various prenatal and perinatal factors associated with PPROM on the incidence of key short and long term neonatal respiratory morbidities—defined as DL, PH, and bronchopulmonary dysplasia (BPD)—as well as on neonatal mortality.

All liveborn infants with PPROM before 32 + 0 weeks and a latency period >13 days who received active neonatal care at one of the participating centers were eligible for inclusion. In accordance with national perinatal guidelines, active postnatal management was considered from 22 + 0 weeks of gestation onward, following individualized interdisciplinary counselling. Pregnancies with major structural fetal anomalies known to independently impair pulmonary development or neonatal survival (e.g., skeletal dysplasia, major congenital heart disease, renal agenesis/Potter sequence) were excluded. Maternal medical and pregnancy-related comorbidities (e.g., diabetes, hypertensive disorders, placental disorders) were not defined as exclusion criteria. These conditions do not directly determine pulmonary hypoplasia or dry lung syndrome, which are primarily influenced by gestational age at rupture, amniotic fluid dynamics, and intrauterine inflammatory exposure.

### Clinical management protocol

Pregnant women presenting with PPROM—regardless of whether the rupture occurred in an outpatient or inpatient setting—underwent standardized clinical evaluation upon admission. PPROM was diagnosed clinically by documented amniotic fluid leakage and confirmed using a placental alpha microglobulin-1 (PAMG-1) immunoassay (AmniSure® test). Diagnosis was based on a positive test result in combination with clinical assessment. Initial assessment included ultrasound examination to determine fetal biometry and document amniotic fluid volume, particularly the single deepest pocket (SDP). Oligohydramnios was defined as a single deepest vertical pocket (SDP) < 2 cm, in accordance with established obstetric ultrasound criteria. The threshold of SDP < 2 cm was chosen *a priori* based on established obstetric ultrasound criteria defining oligohydramnios. This cut-off is widely used in previous PPROM studies (17), providing a clinically interpretable threshold rather than a data-driven *post hoc* categorization. Anhydramnios was defined as absence of a measurable vertical amniotic fluid pocket on ultrasound examination.

Simultaneously, vaginal swabs were obtained for microbiological analysis to detect pathogenic colonization, and maternal laboratory tests (including leukocyte count and C-reactive protein) were performed to assess for signs of systemic infection or inflammation. Cardiotocography (CTG) was employed to evaluate fetal heart rate patterns and overall fetal well-being.

Empirical antibiotic therapy was initiated immediately after diagnosis, consisting of a single 1 g oral dose of azithromycin, followed by 48 h of intravenous ampicillin (2 g, three times daily) and then five days of oral amoxicillin (500 mg, three times daily). Subsequent antibiotic regimens were adjusted based on microbial culture results. The decision to continue inpatient hospitalization vs. transition to outpatient care was made individually, based on maternal and fetal status.

Antenatal corticosteroids for fetal lung maturation were administered according to national guidelines for women with GA > 24 + 0 weeks when PPROM occurred (19). Steroids were not given routinely at the time of PPROM diagnosis in all cases, but were administered when gestational age thresholds were met and/or when clinical assessment indicated an increased likelihood of preterm delivery within the recommended time window. No corticosteroids were given if gestational age was below 23 + 5 weeks or above 33 + 6 weeks. Women with PPROM < 24 weeks were treated with corticosteroids when the pregnancy reached 23 + 5 weeks. This management protocol was consistently applied at both participating centers to ensure standardized care.

### Techniques

Clinical data were obtained as part of routine care during both prenatal and postnatal management. Maternal information was collected from outpatient and inpatient records prior to delivery. Neonatal data were extracted from documentation during the stay in the neonatal intensive care unit (NICU).

Prenatal ultrasound assessments, including measurements of the single deepest pocket (SDP), were performed by board-certified obstetricians specialized in foetal medicine and prenatal diagnostics. All sonographic evaluations adhered to standardized institutional protocols. Data extraction and classification were performed retrospectively from digital patient records and neonatal discharge summaries.

### Data measurements

Data were retrospectively extracted from the institutional electronic medical records (SAP system) of both centers. Maternal and neonatal parameters were evaluated systematically, as outlined below.

### Maternal data

The following maternal parameters were collected:

- GA at the time of PPROM
- Maternal age at PPROM
- Duration of membrane rupture (latency period)
- Minimal and median measurements of the SDP of amniotic fluid
- Administration of antenatal corticosteroids
- Mode of delivery (vaginal vs. caesarean section)
- The maximum leukocyte count recorded around the time of delivery was documented.

Intrauterine inflammation or infection was classified according to German clinical guidelines into three categories (19):

- Triple I (Infection AND Inflammation): maternal fever (>38.0 °C) plus leucocytosis >15,000/µL (without the influence of cortisone due to lung maturation) or positive bacterial vaginal or amniotic fluid culture.
- Inflammation or Infection only: leucocytosis >15,000/µL (without the influence of cortisone due to lung maturation) or positive bacterial culture without fever.
- No signs of inflammation or infection

Vaginal pathogenic bacterial colonization was assessed by vaginal swabs obtained within a maximum duration of 3 days prior to birth and classified into three groups:

- No colonization
- Monomicrobial colonization
- Polymicrobial colonization

### Neonatal data

Neonatal variables included:

- GA at birth
- Sex
- Application of surfactant
- Intraventricular haemorrhage (IVH)

Pulmonary outcomes assessed were:

- Dry Lung Syndrome, defined as reversible postnatal respiratory failure and need of mechanical ventilation with a maximum duration of 48 h
- Pulmonary hypoplasia, defined as ongoing postnatal respiratory failure and need of mechanical ventilation with a duration of more than 48 h
- BPD, classified according to NIH consensus criteria
- Invasive mechanical ventilation (IMV)
- Non-invasive ventilation (NIV)
- Neonatal death

### Pulmonary outcome classification

Pulmonary outcomes were classified into three postnatal respiratory phenotypes: no respiratory distress, transient severe respiratory failure (dry lung phenotype), and persistent severe respiratory failure compatible with pulmonary hypoplasia (PH phenotype).

- Pulmonary hypoplasia (PH phenotype) was defined as persistent postnatal respiratory failure despite standard management for prematurity-related respiratory distress syndrome (RDS), requiring invasive mechanical ventilation beyond 48 h and characterized by clinical features compatible with hypoplastic lung disease following prolonged oligohydramnios. These features included sustained high ventilatory and oxygen requirements, limited radiographic lung expansion with low lung volumes, and/or associated pulmonary hypertension when documented. This pragmatic definition reflects the clinical presentation of PPROM-associated pulmonary hypoplasia as described in the literature, acknowledging that pathological confirmation is rarely available in survivors.
- Dry lung (DL phenotype) was defined as severe initial respiratory failure after prolonged oligohydramnios requiring invasive mechanical ventilation, but with rapid clinical improvement and successful weaning within 48 h. This phenotype is consistent with previously described “dry lung syndrome,” characterized by functional airway collapse and reduced pulmonary fluid dynamics that may clinically mimic hypoplasia but demonstrate early reversibility.

Duration of invasive ventilation (≤48 h vs. >48 h) was therefore used as an operational criterion to distinguish a transient from a persistent respiratory phenotype. We acknowledge that mechanical ventilation duration alone does not constitute a pathological diagnosis of pulmonary hypoplasia and that overlap with severe RDS may occur. The classification should therefore be interpreted as a pragmatic clinical phenotype approach suitable for retrospective analysis rather than a definitive etiologic diagnosis.

### Gestational-age group analyses (PPROM timing and birth gestational age)

To provide a clinically interpretable, group-based description of outcome risks across gestational age, gestational age at PPROM and gestational age at birth were additionally analyzed as ordered categorical variables. Gestational age at PPROM (in days) was binned into four non-overlapping groups reflecting early and later rupture: <23 + 0 weeks, 23 + 0–25 + 6 weeks, 26 + 0–28 + 6 weeks, and ≥29 weeks. Gestational age at birth was binned into six non-overlapping 3-week groups: 24 + 0–26 + 6 weeks, 27 + 0–29 + 6 weeks, 30 + 0–32 + 6 weeks, 33 + 0–35 + 6 weeks, 36 + 0–38 + 6 weeks and >39 weeks. For each binning approach, incidences of dry lung, pulmonary hypoplasia, and death were compared across gestational-age groups using contingency tables.

### SDP evaluation

For each pregnancy, all documented single deepest pocket (SDP) measurements obtained during the latency period were extracted. “Minimal SDP” was defined as the lowest recorded SDP value. “Median SDP” was defined as the within-patient median of all available SDP measurements from PPROM diagnosis until delivery, thereby summarizing the typical amniotic fluid volume over the observation period.

### Group comparison

Given the frequent use of the SDP as a predictive marker, we performed a contingency analysis comparing cases with SDP < 2 cm vs. SDP ≥ 2 cm in relation to the respiratory morbidities DL, PH, BPD and neonatal death.

### Longitudinal SDP evaluation

Serial ultrasound measurements of the SDP were recorded weekly from first presentation after PPROM until delivery. Patients were categorized according to the neonatal respiratory outcome into no respiratory distress, dry lung, or pulmonary hypoplasia. To compare SDP values and their evolution over time between groups while accounting for repeated measurements within the same patient, we analyzed weekly SDP values using a linear mixed-effects model (REML; Satterthwaite df) with Respiratory_Disease_Group (None/Dry lung/pulmonary hypoplasia), PPROM_Week, and their interaction as fixed effects, and an AR(1) covariance structure for within-patient repeated measures. We assessed whether overall SDP levels differed between outcome groups and whether SDP trajectories over time differed between groups. Pairwise group comparisons were adjusted for multiple testing.

### Statistical analysis

Statistical analyses were conducted using SPSS Statistics version 31 (IBM Corp., Armonk, NY) and GraphPad Prism version 10 (GraphPad Software, San Diego, CA). Continuous and ordinal variables were compared using the Mann–Whitney Test, appropriate for the distributional characteristics and scale of the data. Because several cells had small expected counts, Monte-Carlo exact *p*-values (10,000 sampled tables) were used for Pearson Chi^2^ tests. In addition, linear-by-linear association tests were performed to assess monotonic trends across ordered gestational-age categories. To evaluate the risk for the incidence of DL and PH as well as death assess the influence of PPROM associated parameters to these morbidities, a multiple logistic regression model was employed. A *p*-value of <0.05 was considered statistically significant for all analyses.

All multivariable logistic regression models were constructed using a forced-entry (ENTER) approach in SPSS, whereby all predefined maternal and perinatal variables were entered simultaneously. Predictor selection was based on clinical relevance and biological plausibility rather than automated statistical selection procedures.

Multicollinearity was assessed by inspection of correlation matrices and pairwise correlation coefficients between predictors. Correlation coefficients exceeding |0.80| were examined carefully. While moderate correlations were observed (particularly between minimal and median SDP), thresholds indicative of severe collinearity (|r| ≥ 0.90) were generally not exceeded in the DL and LH models. In the mortality model, high intercorrelations and sparse outcome events led to model instability and convergence limitations, which were considered when interpreting results.

## Results

### Study cohort and outcome frequencies

A total of 66 pregnancies/neonates met the inclusion criteria. Maternal, perinatal, and neonatal baseline characteristics, as well as overall frequencies of respiratory outcomes (DL, PH, BPD) and neonatal death, are summarized in Table 1. Maternal age ranged from 19 to 46 years (median 34, IQR 31–37). The distribution of invasive mechanical ventilation (IMV) was markedly skewed, with a median of 0 days (IQR 0–4) but a maximum of 57 days, indicating that while many infants required no IMV, a smaller subgroup experienced prolonged respiratory support. Surfactant was administered according to gestational age and clinical condition using standardized weight-based dosing (100–200 mg/kg). Invasive ventilation was provided as HFOV or assisted-controlled volume-targeted ventilation in accordance with level-III NICU practice. Ventilation modes were not further subclassified, as strategies were individualized and adapted during the clinical course.

**Table 1:** Demographic cohort maternal and neonatal data. Total numbers of the complete cohort on the left.

Maternal data	Total		Neonatal data	Total	
***n*** **=** **66**	Median/*n*	IQR/%	*n* = 66	Median/*n*	IQR/%
Maternal Age (years)	34	31–37	GA at Birth (week/day)	31/1	27/3–34/1
GA at PPROM (week/day)	24/4	22/5–27/6	Sex (♂/♀)	35/31	53.0/47.0
Duration PPROM (days)	38	22–56	Surfactant yes	44	66.7
Minimal SDP (cm)	1.8	1.1–2.8	IMV (days)	0	0–4.0
Median SDP (cm)	2.9	2.2–4.3	NIV (days)	5.0	0–36.0
Maximum Leucocytes (/nl)	13	10–15	Dry Lung	14	21.2
Spontaneous/Caesarean Section	15/51	23/77	Pulmonary Hypoplasia	14	21.2
Antenatal Steroids			Death	7	10.6
None	8	12,1	BPD (*n* = 59)		
1 Dose	0	0	None	47	79.6
2 Doses	55	83.3	Mild	8	13.6
Recue dose	3	4.6	Moderate	2	3.4
Infection/Inflammation			Severe	2	3.4
None	41	62.1	IVH		
Infection or Inflammation	19	28.8	None	57	86.3
Triple I	6	9.1	I°	3	4.6
Pathogenic vaginal Microbiome at Birth			II°	6	9.1
None	33	50.0	III°	0	0
Single Strain	18	27.3			
Multiple Strains	15	22.7			

GA, gestational age; IQR, interquartile range; PPROM, preterm premature rupture of membranes; SDP, single deepest pocket; BPD, bronchopulmonary dysplasia; IVH, intra ventricular haemorrhage; OR, odds ratio; IMV, invasive mechanical ventilation; NIV, non-invasive ventilation.

### Gestational age group analyses

To provide an interpretable risk overview across clinically ordered gestational-age strata, outcomes were evaluated across GA at PPROM- and GA at birth-groups (Table 2). Across GA at PPROM groups, the earlier the rupture we observed associated higher rates of PH, death, and BPD, with significant ordered trends and moderate to large association (Cramer's V: PH 0.46/Death 0.42/BPD 0.53); DL did not show a significant association or trend across PPROM timing.

**Table 2:** Association between gestational age strata (at PPROM and at birth) and neonatal respiratory outcomes. The upper section of the table presents outcomes stratified by gestational age at PPROM, while the lower section shows outcomes stratified by gestational age at birth. Respiratory outcomes (dry lung, pulmonary hypoplasia, death, and BPD) are displayed as *n* (%) within each gestational-age group. Group differences were assessed using Pearson Chi^2^ tests with Monte-Carlo exact *p*-values; ordered trends across increasing gestational-age categories were evaluated using linear-by-linear association testing. Effect sizes are reported as Cramer's V.

	Outcome *n* = (%)				Association Testing			
GA at PPROM	Dry Lung	Pulmonary Hypoplasia	Death	BPD	Outcome	Chi^2^ *p*=	Chi^2^ Trend *p*=	Cramer's V Effect Size
(*n* = 66/BPD *n* = 59)	*n* = / %	*n* = / %	*n* = / %	*n* = / %				
<22 + 6 weeks (*n* = 20/BPD *n* = 14)	5 (25)	9 (45)	6 (30)	8 (57)				
23 + 0–25 + 6 weeks (*n* = 20/BPD *n* = 19)	3 (15)	5 (25)	1 (5)	2 (11)				
26 + 0–28 + 6 weeks (*n* = 13/BPD *n* = 13)	5 (38)	0 (0)	0 (0)	2 (15)				
29 + 0–31 + 6 weeks (*n* = 13/BPD *n* = 13)	1 (8)	0 (0)	0 (0)	0 (0)				
Total (*n* = 66/BPD *n* = 59)	14 (21)	14 (21)	7 (17)	12 (20)				
Overall test					Dry Lung	0.22	0.59	0.26
					Pulmonary Hypoplasia	**	***	0.46
					Death	*	**	0.42
					BPD	**	***	0.53
GA at Birth	Outcome *n* = (%)				Association Testing			
(*n* = 66/BPD *n* = 59)	Dry Lung	Pulmonary Hypoplasia	Death	BPD	Outcome	Chi^2^ *p*=	Chi^2^ Trend *p*=	Cramer's V Effect Size
	*n* = / %	*n* = / %	*n* = / %	*n* = / %				
24 + 0–26 + 6 weeks (*n* = 14/BPD *n* = 12)	3 (21)	6 (43)	2 (14)	8 (67)				
27 + 0–29 + 6 weeks (*n* = 15/BPD *n* = 12)	5 (33)	5 (33)	3 (20)	2 (17)				
30 + 0–32 + 6 weeks (*n* = 11/BPD *n* = 9)	2 (18)	3 (27)	2 (18)	1 (11)				
33 + 0–35 + 6 weeks (*n* = 17/BPD *n* = 17)	4 (24)	0 (0)	0 (0)	0 (0)				
36 + 0–38 + 6 weeks (*n* = 6/BPD *n* = 6)	0 (0)	0 (0)	0 (0)	0 (0)				
>39 + 0 weeks (*n* = 3/BPD *n* = 3)	0 (0)	0 (0)	0 (0)	1 (33)				
Total (*n* = 66/BPD *n* = 59)	14 (21)	14 (21)	7 (11)	12 (20)				
Overall test					Dry Lung	0.60	0.26	0.24
					Pulmonary Hypoplasia	*	***	0.44
					Death	0.37	0.09	0.29
					BPD	**	**	0.62

GA, gestational age; BPD, bronchopulmonary dysplasia.

*p < 0.05.

**p < 0.01.

***p < 0.001.

Across GA at birth groups, lower GA at birth was associated with higher rates of PH and BPD, with significant trends across increasing gestational-age strata and with significant ordered trends and moderate to large association (Cramer's V: PH 0.44/BPD 0.62) as well, whereas DL did not differ significantly across birth-GA groups; death showed no statistically significant overall association across birth-GA groups in this categorical analysis.

### Multiple logistic regression analysis

Regression models are detailed in Table 3. These identified several significant predictors of respiratory morbidity and mortality:

- Maternal age was significantly associated with PH (*p* < 0.05) and neonatal death (*p* < 0.001), suggesting its broader relevance in pregnancy risk stratification.
- GA at PPROM showed significant associations PH and death except DH and BPD, with the strongest link to death (*p* < 0.01) matching the results from the group comparison.
- Minimal SDP and median SDP were significantly associated with BPD (*p* < 0.05). Minimal SDP also showed a strong non-significant trend for death (*p* = 0.07).
- Inflammation/infection was a significant positive predictor of BPD (*p* < 0.05) and appeared related to neonatal death (*p* < 0.01).
- Vaginal colonization with multiple pathogens was significantly associated with neonatal death (*p* < 0.05), although no significant difference between groups was seen in bivariate comparison. Single pathogenic strains seem to influence the occurrence of DL (*p* < 0.05).

**Table 3:** Multiple logistic regression for neonatal respiratory morbidities and death.

Risk Factor	Dry Lung	Pulmonary Hypoplasia	Death	BPD
	*p*-Value/OR (CI 95%)	*p*-Value/OR (CI 95%)	*p*-Value/OR (CI 95%)	*p*-Value/OR (CI 95%)
	*r*^2^ = 0.15	*r*^2^ = 0.44	*r*^2^ = 0.65	*r*^2^ = 0.71
Maternal Age	0.91/0.99 (0.86–1.14)	*/1.19 **(1.02–1.46)**	***/2.12 **(1.31–6.7)**	0.20/0.75 (0.33–1.13)
GA PPROM in Days	0.31/1.02 (0.98–1.06)	*/1.05 **(1.01–1.12)**	**/1.22 **(1.04–1.68)**	0.14/1.07 (0.98–1.21)
Length PPROM in days	0.55/1.01 (0.98–1.05)	0.37/1.02 (0.98–1.07)	0.83/0.99 (0.92–1.07)	0.13/1.05 (0.99–1.16)
SDP Minimum	0.97/1.01 (0.5–2.17)	0.40/2.20 (0.38–18.5)	0.07/85 (0.7–734,675)	*/28.2 **(1.26–**5,614**)**
SDP Median	0.86/0.93 (0.42–2.17)	0.71/1.33 (0.31–6.96)	0.07/0.03 (0.008–1.22)	0.18/4.4 (0.55–165)
Max. Leucocytes (/nl)	0.76/1.02 (0.89–1.21)	0.67/0.98 (0.82–1.14)	0.82/0.96 (0.66–1.61)	0.38/1.12 (0.88–1.56)
Infection OR Inflammation	0.74/1.34 (0.25–8.71)	0.64/1.61 (0.22–13.6)	**/0.15 **(0.05–0.53)**	*/87.2 **(1.58–547,032**)
Triple I	0.68/0.64 (0.07–5.8)	0.72/1.79 (0.09–93.8)	0.21/0.19 (0.02–1.5)	0.81/1.82 (0.01–3,046)
Pathogenic Microbiome Single	*/0.17 **(0.03–0.85)**	0.56/1.82 (0.25–16.3)	0.33/0.04 (6,1^−8^–18.5)	0.08/90 (0.6–41,70,669)
Pathogenic Microbiome Multiple	0.20/0.31 (0.05–1.8)	0.07/9.71 (0.8–213)	0.11/0.02 (3,96^−7^–2.0)	0.22/10.1 (0.3–2,392)

GA, gestational age; PPROM, preterm premature rupture of membranes; SDP, single deepest pocket; BPD, bronchopulmonary dysplasia.

* *p* < 0.05.

** *p* < 0.01.

*** *p* < 0.001.

**Figure 1:**
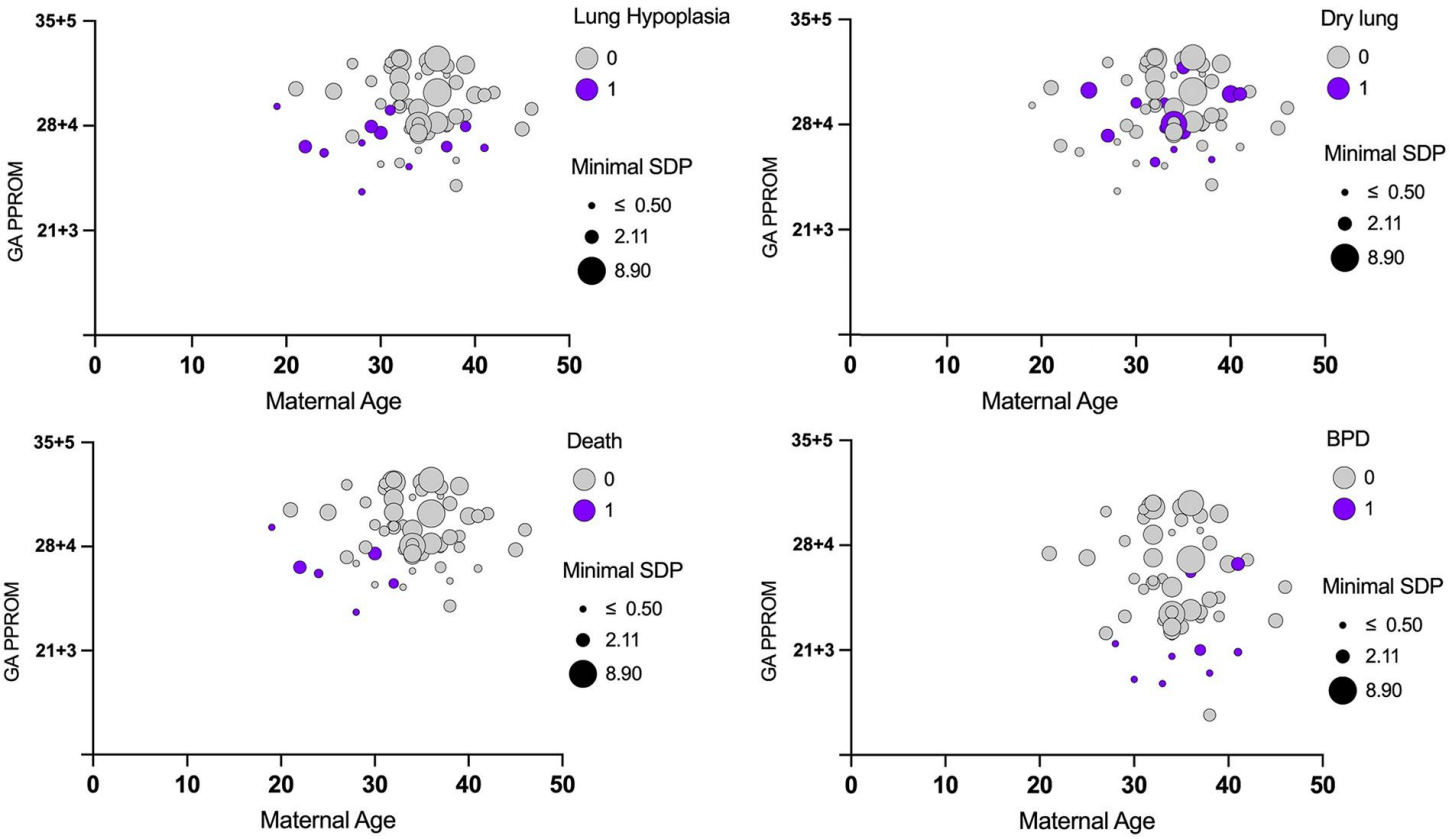
Correlations of the most relevant influencing parameters (see Table 3) GA at PPROM (in weeks), the maternal age (in years) at birth and the minimal SDP for short- and long-term respiratory outcomes. Dot size resembles the amount of SDP in cm. 0 = No, 1 = Yes; GA, gestational age; SDP, single deepest pocket.

**Figure 2:**
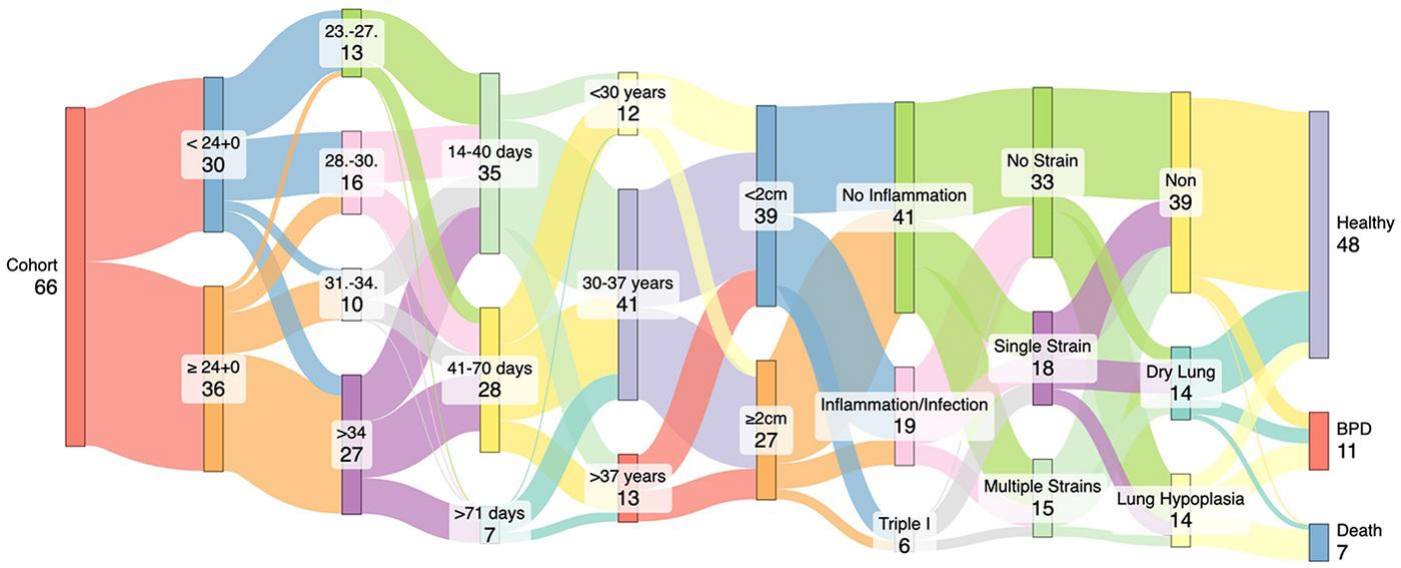
Sankey-diagram showing trajectories of relevant influencing maternal and neonatal influencing factors. This illustrates the complexity of interactions of prenatal influencing factors on postnatal outcome. GA, gestational age; SDP, single deepest pocket; PPROM, preterm premature rupture of membranes.

Model R^2^ values indicated moderate to high explanatory power, ranging from 0.15 (dry lung) to 0.71 (BPD). The correlation of the most relevant results (Maternal Age, SDP and GA at PPROM) concerning the respiratory morbidities are plotted in Figure 1. Trajectories of patient characteristics are shown in the Sankey-Diagram in Figure 2.

In crude outcome frequencies, neonatal death occurred in 3/41 (7.3%) without inflammation, 3/19 (15.8%) with infection/inflammation, and 1/6 (16.7%) with Triple I. BPD occurred in 9/41, 2/19, and 1/6 cases, respectively. Interpretation of the Triple I subgroup is limited by small numbers (*n* = 6). Descriptive leukocyte values presented in Table 1 reflect overall cohort distribution and were not used as continuous predictors of inflammation status.

### SDP cut-off and longitudinal development evaluation

A median SDP < 2 cm was observed in *n* = 13/66 (19,7%) pregnancies, while a minimal SDP < 2 cm occurred in *n* = 27/66 (40,9%) cases. Contingency analyses demonstrated that adverse outcomes were disproportionately represented in the median SDP < 2 cm group, particularly for lung hypoplasia, bronchopulmonary dysplasia, and neonatal death. The duration aspect of oligohydramnios was addressed through longitudinal trajectory evaluation using serial SDP measurements across the latency period.

As shown in Table 4, subgroup analysis using an SDP threshold of 2 cm demonstrated clear clinical relevance:

- Median SDP < 2 cm was significantly associated with PH (*p* < 0.001), BPD (*p* < 0.001), and neonatal death (*p* < 0.05).
- Minimal SDP < 2 cm also correlated significantly with PH (*p* < 0.01), but not with death or DL.

**Table 4:** Comparison of SDP measurements < or ≥ than 2 cm for minimum or median correlated to neonatal respiratory outcome and death.

SDP-Comparison	Dry Lung	Pulmonary Hypoplasia	Death	BPD
	*p*-Value/OR (CI 95%)	*p*-Value/OR (CI 95%)	*p*-Value/OR (CI 95%)	*p*-Value/OR (CI 95%)
SDP Minimum < vs. ≥2 cm	0.55/1.6 (0.45–5.0)	**/13 **(2.0–143)**	0.23/4.7 (0.67–56)	*** **/** **(** **1.26–5,614)**
SDP Median < vs. ≥2 cm	0.30/2.1 (0.61–6.9)	***/13 **(3.0–56)**	*/7.4 **(1.7–32)**	***/21 **(4.4–84)**

GA, gestational age; PPROM, preterm premature rupture of membranes; SDP, single deepest pocket; BPD, bronchopulmonary dysplasia.

* *p* < 0.05.

** *p* < 0.01.

*** *p* < 0.001

Across the observation period, SDP values were significantly lower in pregnancies resulting in pulmonary hypoplasia compared with those without respiratory distress, whereas dry lung cases did not significantly differ from either group. These findings support the use of SDP < 2 cm as a prognostic marker for adverse pulmonary outcomes following PPROM.

**Figure 3:**
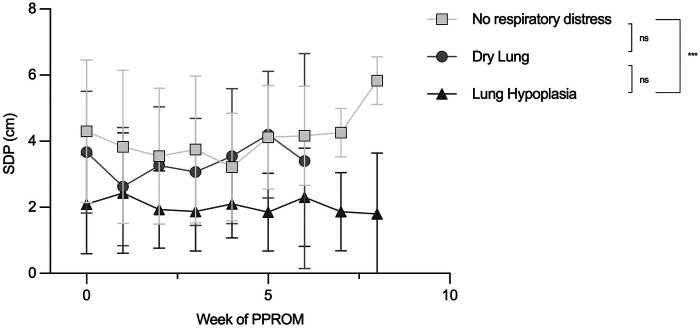
Trajectories of SDP measurements for patients according to their respiratory outcome with mean and SEM plots. Significant difference in overall SDP measurement between patients without respiratory distress against patients with Pulmonary Hypoplasia outcome. SDP, single deepest pocket; PPROM, preterm premature rupture of membranes; SEM, standard error of mea.

Overall, patients with pulmonary hypoplasia had consistently lower SDP values compared with those with no respiratory distress (pairwise comparison *p* < 0.001). Differences between dry lung and no respiratory distress were not significant (*p* = 0.271), and the comparison between dry lung and pulmonary hypoplasia did not reach statistical significance after correction (*p* = 0.066). Importantly, there was no evidence that SDP changed systematically over the weeks in the cohort (*p* = 0.521) and no evidence that the SDP course over time differed between outcome groups (no group-by-time interaction; *p* = 0.854). Thus, the main difference between groups was a persistent separation in SDP level (lowest in pulmonary hypoplasia), rather than diverging or converging trajectories during follow-up. Trajectories can be seen in Figure 3.

## Discussion

Our study examined the multifactorial risk profile of pregnancies complicated by prolonged second-trimester PPROM, with particular attention to neonatal pulmonary outcomes. The findings highlight the persistent challenges in managing PPROM in midgestation, where balancing latency extension to improve maturity is complicated by the rising risks of pulmonary hypoplasia, dry lung syndrome, and perinatal death.

### Gestational age at PPROM and birth: critical determinants of outcome

According to our findings gestational age at PPROM is a fundamental determinant of neonatal respiratory outcomes, with earlier rupture associated with significantly higher rates of pulmonary hypoplasia. These associations align with extensive literature demonstrating that previable PPROM (<24 weeks) carries substantially higher morbidity and mortality compared to later gestational ages (25–27). Increased gestational age at rupture was associated with improved neonatal survival consistent with our findings (28). The biological plausibility of this relationship is well-established: PPROM occurring during the canalicular phase of lung development (16–28 weeks) disrupts critical alveolarization and vascular development processes, leading to structural pulmonary hypoplasia (25, 29). Stratified analysis by gestational age at birth demonstrated significant ordered associations for pulmonary hypoplasia and BPD, with decreasing morbidity across increasing gestational-age categories. This dual effect—both timing of rupture and timing of delivery—underscores the complex interplay between the duration of oligohydramnios exposure and the degree of prematurity at birth. Neonatal mortality was reported of 63.2% in the 23–27 + 6-week gestational age group compared to 2.2% in the 34–36 + 6-week group, highlighting the critical importance of achieving later gestational ages at delivery (30).

### The SDP < 2 cm threshold: a clinically actionable biomarker

One of the most clinically significant findings of our study is the identification of SDP < 2 cm as a robust predictor of adverse outcomes. An SDP < 2 cm was significantly associated with pulmonary hypoplasia, BPD and death. These findings provide a specific, measurable threshold that can be readily applied in clinical practice using standard ultrasound techniques. The longitudinal SDP analysis further strengthened these findings, demonstrating that neonates who developed pulmonary hypoplasia had consistently lower SDP measurements throughout the latency period compared to those without respiratory distress with no significant change over time. These findings indicate that persistently lower absolute SDP levels, rather than differential changes over time, are associated with lung hypoplasia. The absence of a significant group-by-time interaction suggests that sustained oligohydramnios level—rather than divergent trajectories during follow-up—may be the more relevant determinant of pulmonary development in this cohort. Our findings align with previous research linking severe oligohydramnios to adverse outcomes. Anhydramnios combined with low gestational age at PPROM negatively influenced neonatal outcomes, including pulmonary hypoplasia and prolonged anhydramnios after PPROM was associated with a four-fold increased risk of composite adverse outcomes, including death and BPD (11, 29). However, few studies have established specific quantitative thresholds for clinical decision-making. The 2 cm threshold identified in our study provides a practical cutpoint for risk stratification and counseling. A maximum vertical pocket (MVP) of amniotic fluid of <1 cm as a criterion for severe oligohydramnios in twin pregnancies with PPROM as predictor for mortality was not significant (31). The discrepancy may reflect differences in measurement techniques (MVP vs. SDP) and population characteristics. Our use of SDP, which is more commonly employed in clinical practice, enhances the generalizability of our findings. The biological mechanism underlying the SDP-outcome relationship is multifactorial. Severe oligohydramnios restricts fetal breathing movements, which are essential for lung growth and development (25). Additionally, reduced amniotic fluid volume leads to chest wall compression and decreased intrathoracic space, further impairing pulmonary expansion. The consistency of low SDP measurements over time in our pulmonary hypoplasia group suggests that sustained mechanical restriction, rather than transient oligohydramnios, drives the development of structural pulmonary abnormalities. While latency duration was included as a covariate, the precise cumulative duration of oligohydramnios (e.g., total weeks with SDP < 2 cm) was not separately quantified. However, serial weekly SDP measurements allowed assessment of persistent fluid depletion patterns. Our findings suggest that sustained low absolute SDP levels—rather than latency alone—were associated with lung hypoplasia. Not all pregnancies with PPROM demonstrated sustained severe oligohydramnios; variability in serial SDP measurements was observed, underscoring the distinction between membrane rupture and actual fluid depletion. Median SDP likely reflects sustained amniotic fluid conditions across the latency period, whereas the minimum SDP may capture brief nadirs caused by measurement variability, transient fetal position effects, or short episodes of more severe fluid depletion. In this cohort, associations with adverse outcomes were stronger for median SDP < 2 cm, consistent with the interpretation that persistent oligohydramnios—rather than isolated low values—may be more relevant for chronic pulmonary compromise and downstream morbidity. Nevertheless, given the limited event numbers and wide confidence intervals, we cannot exclude that differences between minimum and median SDP associations may partly reflect statistical imprecision.

### Maternal age: an underappreciated risk factor

In our multivariable analysis, maternal age was statistically associated with lung hypoplasia and neonatal death. Although the median maternal age of 34 years lies within the contemporary reproductive range, the distribution in our cohort (19–46 years) reflects the current demographic trend toward delayed childbearing. Maternal age was analysed as a continuous variable and the association persisted after adjustment for other covariates; however, given the modest sample size and the width of confidence intervals, this finding should be interpreted cautiously. We therefore consider maternal age in this cohort as a potential contributory factor rather than a causal determinant. Larger studies are required to clarify whether this observation represents a reproducible biological signal or reflects residual confounding.

### The inflammation/infection aspect

Another intriguing finding of our study is the relationship between inflammation/infection and outcomes. Markers of inflammation/infection were positively associated with BPD and related to death, suggesting that inflammatory processes increase the risk of chronic lung disease. Intrauterine inflammation and infection trigger fetal inflammatory responses that accelerate lung maturation through increased surfactant production and structural maturation. Early neonatal infection increased the risk of respiratory distress syndrome immensely and causes ongoing lung injury, disrupting alveolarization and vascular development, ultimately leading to BPD (25, 26). BPD can occur in up to three quarters of surviving neonates after previable PPROM, with significantly higher rates in those with longer latency periods, suggesting cumulative inflammatory exposure (28). The chronic inflammatory milieu associated with prolonged PPROM may thus represent a “double-edged sword”—enhancing immediate survival at the cost of long-term pulmonary morbidity. Our findings have important implications for the management of PPROM. While antibiotic prophylaxis is standard to prevent overt infection, the optimal balance between preventing infection-related mortality and minimizing inflammation-driven BPD remains unclear. Antibiotic use reduces respiratory distress syndrome incidence, supporting current practice guidelines (25). However, the long-term pulmonary consequences of prolonged antibiotic exposure and the potential for selecting resistant organisms warrant consideration. The association between maternal inflammation and adverse neonatal outcomes should be interpreted cautiously. While crude frequencies showed variability across inflammatory categories, the Triple I subgroup was small, limiting statistical power. The observed associations were derived from multivariable models adjusting for gestational age, latency, and amniotic fluid parameters, suggesting that inflammatory exposure may act in conjunction with prematurity-related factors rather than as an isolated determinant. These findings should therefore be considered exploratory and hypothesis-generating.

### Microbiome and colonization patterns: emerging predictors

In the adjusted analysis, vaginal colonization patterns demonstrated a nuanced relationship with neonatal outcomes. Single-strain pathogenic colonization was independently associated with lower odds of dry lung, whereas polymicrobial colonization did not show a statistically significant association with respiratory morbidity or death. These findings suggest that maternal microbiome patterns may interact with neonatal respiratory adaptation in complex ways; however, given the limited sample size and wide confidence intervals, these observations should be interpreted cautiously (25). Chorioamnionitis rates were significantly higher in mothers of non-surviving neonates, supporting the link between infection and mortality (28). While infection and inflammation have been implicated in adverse neonatal outcomes in previous studies, our data do not support a direct independent effect of polymicrobial colonization on mortality in this cohort. The observed association between single-strain colonization and dry lung may reflect differences in inflammatory exposure or pathogen–host interactions, but this remains speculative. Given that dry lung represents functional respiratory instability rather than structural pulmonary hypoplasia, the underlying mechanisms may differ from those driving chronic lung injury. Further studies incorporating detailed microbial characterization and inflammatory biomarkers are required to clarify these relationships.

Maternal vaginal colonization in this study was interpreted as a marker of potential intrauterine inflammatory exposure rather than as evidence of microbiologically confirmed neonatal infection but these findings underscore the potential value of comprehensive microbiome assessment in PPROM management. Current practice typically focuses on Group B Streptococcus screening and broad-spectrum antibiotic prophylaxis, but more targeted approaches based on specific colonization patterns may improve outcomes. Future research should investigate whether microbiome-directed therapies can reduce adverse outcomes in PPROM pregnancies.

### Distinguishing dry lung syndrome from pulmonary hypoplasia: clinical and prognostic implications and model performance

Our study distinguished between dry lung phenotype and pulmonary hypoplasia phenotype as separate outcome categories, an important differentiation that is often overlooked in the PPROM literature. While both conditions present with respiratory distress, their underlying pathophysiology, clinical course, and prognosis differ substantially. Pulmonary hypoplasia represents true structural underdevelopment of the lungs, with reduced alveolar number, simplified architecture, and abnormal vascular development. This condition typically results from prolonged severe oligohydramnios during critical periods of lung development and carries a high mortality rate (11, 26). In our study, pulmonary hypoplasia was strongly associated with earlier gestational age at PPROM, severe oligohydramnios and maternal age. In contrast, dry lung syndrome represents functional airway collapse due to reduced lung fluid production and altered surfactant dynamics, without necessarily involving structural hypoplasia. This distinction is further reflected in the ventilation data of our cohort. Although the overall median duration of invasive mechanical ventilation was 0 days, reflecting that many infants required no IMV, a subset—particularly those with pulmonary hypoplasia—required prolonged support (maximum 57 days). This skewed distribution aligns with the concept that pulmonary hypoplasia is characterized by sustained ventilatory dependency, whereas extreme prematurity without structural impairment may require only short-term stabilization. This condition may show more rapid improvement with appropriate ventilatory support and surfactant administration. Our model for dry lung had a lower R² (0.15), suggesting that this condition is less predictable from prenatal factors and may be more influenceable by postnatal management strategies. The distinction between these conditions has important implications for prenatal counseling and postnatal management. Families facing a diagnosis of suspected pulmonary hypoplasia should be counseled about the high risk of mortality and severe long-term morbidity, while those with suspected dry lung may have a more favorable prognosis. Antenatal differentiation between these conditions remains challenging, as both may present with similar ultrasound findings. However, the severity and duration of oligohydramnios, gestational age at rupture, and chest circumference measurements may provide clues.

The predictive models developed in our study demonstrated substantial explanatory power, with R² values of 0.15 for dry lung, 0.44 for pulmonary hypoplasia, 0.65 for death, and 0.71 for BPD. These values indicate that our models explain a meaningful proportion of outcome variability, particularly for BPD and mortality. The high R² for BPD (0.71) suggests that prenatal and perinatal factors captured in our study—including gestational age, SDP measurements, and inflammation/infection markers—are strong predictors of chronic lung disease. These findings suggest that selected prenatal parameters, particularly gestational age at rupture and longitudinal amniotic fluid assessment, may contribute to identifying infants at higher risk for BPD. However, given the limited number of BPD cases in this cohort (*n* = 12, with 4 moderate/severe), the present analysis should not be interpreted as a validated predictive model. No formal predictive performance metrics (e.g., discrimination or calibration analyses) were calculated. Rather, these results provide exploratory evidence that certain prenatal factors are associated with BPD and warrant confirmation in larger, prospectively designed studies before clinical risk stratification algorithms can be recommended. The moderate R² for pulmonary hypoplasia (0.44) indicates that while our model captures important predictive factors, substantial variability remains unexplained. This may reflect the complex, multifactorial nature of pulmonary development and the influence of factors not captured in our study, such as genetic predisposition, placental function, and fetal growth patterns. The lower R² for dry lung (0.15) suggests that this condition is particularly difficult to predict prenatally and may be more dependent on postnatal factors and management strategies. The strong predictive performance for mortality (R² = 0.65) is clinically valuable for prenatal counseling. Families facing PPROM at previable or periviable gestational ages require accurate prognostic information to make informed decisions about pregnancy management. Our findings suggest that a combination of gestational age at PPROM, SDP measurements, maternal age, and colonization patterns can provide meaningful risk stratification for mortality. Potential collinearity between gestational-age–related and sonographic variables was formally assessed prior to model construction. No critical multicollinearity was identified, permitting simultaneous inclusion of these predictors in the multivariable analyses. Nevertheless, given the limited sample size and wide confidence intervals for selected variables, particularly infection- and microbiome-related parameters, individual effect estimates should be interpreted with caution and warrant validation in larger cohorts.

### Limitations

Several limitations should be acknowledged. The retrospective design limits causal inference and may introduce selection bias. Patients who continued pregnancy after PPROM represent a selected population, as some may have chosen termination or experienced early pregnancy loss. The relatively small sample size (*n* = 66) limits statistical power for detecting modest associations and precludes extensive subgroup analyses. Maternal comorbidities were not systematically captured as structured variables in this retrospective dataset and were therefore not included in multivariable adjustment. Although such conditions may influence prematurity-related risk, they are not primary determinants of pulmonary hypoplasia or dry lung syndrome. Moreover, given the limited number of outcome events—particularly for neonatal death—the number of covariates included in regression models had to be restricted to avoid model overfitting and unstable estimates. Residual confounding cannot be excluded. Although such conditions may influence placental function and amniotic fluid dynamics, residual confounding cannot be excluded. Birth weight was not included in multivariable models because of its strong correlation with gestational age at birth and its position on the causal pathway between prematurity and respiratory outcome, which may introduce over adjustment bias. Therefore, gestational age was retained as the primary maturity-related covariate. The study period may have spanned changes in clinical practice that could influence outcomes. The long-term neurodevelopmental outcomes were not assessed, limiting our understanding of the full impact of PPROM and its complications. Classification of dry lung and pulmonary hypoplasia was based on standardized clinical documentation and postnatal respiratory course rather than pathological confirmation or advanced imaging. Given the overlap between severe prematurity-related RDS, functional airway collapse, and true pulmonary hypoplasia in extremely preterm infants, some degree of misclassification cannot be excluded. We therefore interpret DL and PH as pragmatic postnatal respiratory phenotypes suitable for retrospective analysis rather than definitive etiologic diagnoses. Although collinearity was formally assessed and no critical interdependence was detected, the limited sample size and low event numbers in certain categories may have affected coefficient stability and widened confidence intervals in multivariable models.

## Conclusion

This study provides important insights into the prediction of respiratory outcomes and mortality in neonates born after PPROM before 32 weeks with prolonged latency. The identification of SDP < 2 cm as a clinically actionable threshold, the recognition of maternal age as a significant risk factor, and the characterization of the inflammation/infection contribute to our understanding of this complex condition. The observed associations between prenatal parameters and neonatal outcomes highlight potentially relevant factors for risk awareness and counselling. However, given the limited sample size and absence of formal predictive validation, these findings should be considered exploratory.

Our findings underscore the multifactorial nature of outcomes after PPROM, involving the interplay of gestational age, amniotic fluid volume, maternal factors, and infection/inflammation. The distinction between dry lung syndrome and pulmonary hypoplasia highlights the heterogeneity of respiratory morbidity in this population and the need for individualized assessment and management.

While PPROM before 32 weeks with prolonged latency remains a high-risk condition associated with substantial morbidity and mortality, our study demonstrates that meaningful risk stratification is possible using readily available clinical parameters. These findings can inform prenatal counseling, guide monitoring strategies, and potentially identify targets for intervention to improve outcomes in this vulnerable population. Future research should focus on validating these findings in larger, prospective cohorts and evaluating interventions to reduce the burden of respiratory morbidity and mortality in PPROM pregnancies. Prospective studies with larger cohorts are required to determine whether these variables can be integrated into a clinically applicable risk stratification model.

## Data Availability

The data analyzed in this study is subject to the following licenses/restrictions: In the context of self-research, recording as well as storage of all personal data already known through treatment in pseudonymized form in a database in the network of the clinic for 10 years (§ 630f BGB). No passing on of data to third parties. No data will be passed on to persons who are not part of the study team. Guarantee of data security by exclusive use of the clinic network as well as securing the database by means of a password known only to the study team. Publication of data exclusively in anonymized form. The datasets generated during and/or analyzed during the current study are available from the corresponding author on reasonable request. Requests to access these datasets should be directed to Dr. Christian Brickmann, christian.brickmann@mri.tum.de.
