# Supplementary material for: PROMISE—impact of maternal and neonatal risk factors on the respiratory outcome of extremely preterm infants following PPROM in the second trimester of pregnancy
Source: Front Pediatr. 2026 Apr 8;14:1776970. doi: 10.3389/fped.2026.1776970 (PMC13099901; doi:10.3389/fped.2026.1776970)
Supplement: Supplementary file 1 [file Table1.docx]

**Supplemental material:**

Supplemental Table 1: Distribution of neonatal respiratory outcomes according to maternal inflammatory status. Frequencies of dry lung (DL), pulmonary hypoplasia (PH), neonatal death, and bronchopulmonary dysplasia (BPD) are presented across three maternal categories: no inflammation, Infection/inflammation, and Triple I. Outcomes are reported as absolute counts within each inflammatory group. Respiratory diagnoses (DL and PH) were mutually exclusive; death and BPD could co-occur with respiratory outcomes.

|  | None (n=38/66)  n= / (%) | Dry Lung (n=14/66)  n= / (%) | Pulmonary Hypoplasia (n=14/66)  n= / % | Death (n=7/66)  n= / % | BPD (n=12/59)  n= / % |
| --- | --- | --- | --- | --- | --- |
| None (n=41/66) | 25 | 8 | 8 | 3 | 9 |
| Infection / Inflammation (n=19/66) | 8 | 3 | 5 | 3 | 2 |
| Triple I (n=6/66) | 8 | 3 | 1 | 1 | 1 |
